# Population pharmacokinetics of pyrazinamide and isoniazid in plasma and cerebrospinal fluid from South African adults with tuberculous meningitis

**DOI:** 10.1128/aac.00099-25

**Published:** 2025-07-01

**Authors:** Jose M. Calderin, Sean Wasserman, Juan Eduardo Resendiz-Galvan, Noha Abdelgawad, Angharad Davis, Cari Stek, Lubbe Wiesner, Robert J. Wilkinson, Paolo Denti

**Affiliations:** 1 Division of Clinical Pharmacology, Department of Medicine, University of Cape Town 71984, Cape Town, Republic of South Africa; 2 Wellcome Discovery Research Platforms in Infection, Centre for Infectious Diseases Research in Africa, Institute of Infectious Disease and Molecular Medicine, University of Cape Town 71985, Cape Town, Republic of South Africa; 3 Institute for Infection and Immunity, City St George’s, University of London 244882, London, United Kingdom; 4 The Francis Crick Institute 376570, London, United Kingdom; 5 Faculty of Life Sciences, University College London 98551, London, United Kingdom; 6 Division of Infectious Diseases and HIV Medicine, Department of Medicine, University of Cape Town 575383, Cape Town, Republic of South Africa; 7 Department of Infectious Diseases, Imperial College London 170895, London, United Kingdom; University of California San Francisco, San Francisco, California, USA

**Keywords:** population pharmacokinetics, tuberculous meningitis, pyrazinamide, isoniazid, NAT2 phenotype, cerebrospinal fluid penetration, high-dose rifampicin, nonlinear mixed-effects modeling, NONMEM

## Abstract

Pyrazinamide and isoniazid are first-line drugs for tuberculous meningitis (TBM), but limited information is available on their plasma pharmacokinetics, and particularly cerebrospinal fluid (CSF) penetration, in patients with TBM. Any potential effect of co-administration with high-dose rifampicin, also being evaluated in trials for TBM, is unknown. Understanding this is important for dose optimisation. We characterized pyrazinamide and isoniazid plasma and CSF pharmacokinetics among adults enrolled in a phase 2 clinical trial of intensified antibiotic therapy for HIV-associated TBM. Participants were randomized to receive either standard TBM treatment (including rifampicin 10 mg/kg) or high-dose rifampicin (35 mg/kg) plus linezolid, with or without aspirin. Plasma and lumbar CSF samples were collected on days 3 and 28 after study enrollment, and drug concentrations were measured using liquid chromatography-tandem mass spectrometry. Data were analysed using nonlinear mixed-effects modeling. Forty-nine participants provided 414 plasma and 44 CSF concentrations. Pyrazinamide CSF concentrations equilibrated with plasma with a half-life of 0.66 h and a pseudo-partition coefficient of 1.05. Isoniazid concentrations equilibrated with a half-life of 3.87 h and a pseudo-partition coefficient of 1.04. Pyrazinamide clearance increased by 30% from day 3 to day 28. NAT2 phenotype determined multi-modal isoniazid clearance. High-dose rifampicin did not affect pyrazinamide or isoniazid plasma pharmacokinetics or CSF penetration. Both drugs achieved exposure in CSF similar to plasma, supporting their crucial role in TBM treatment. Plasma pharmacokinetics of pyrazinamide and isoniazid in TBM were consistent with previously reported values in pulmonary tuberculosis, even when co-administered with high-dose rifampicin.

## INTRODUCTION

Tuberculous meningitis (TBM) is the most serious and debilitating form of tuberculosis (TB). Treatment outcomes are poor, especially among people with HIV, where mortality can exceed 50%, and a third of survivors experience chronic neurological impairment (1).

International guidelines recommend the same drug regimen and dosing as for drug-susceptible pulmonary TB (1). However, the movement of drugs between the systemic circulation and the cerebrospinal fluid (CSF) is restricted by the blood-brain barrier (BBB) and the blood-CSF barrier (2). These barriers may limit the penetration of drugs into the central nervous system (CNS), leading to subtherapeutic exposures at the site of the disease in TBM, potentially contributing to poor outcomes. Additionally, disease-related factors, such as changes in BBB permeability and CSF protein concentrations could affect drug exposure to CSF, potentially impacting efficacy (3, 4).

Pyrazinamide and isoniazid are key first-line drugs for TB due to their potent activity against *Mycobacterium tuberculosis* and their contribution to treatment shortening (5). Both drugs are rapidly absorbed after oral administration, reaching peak concentrations within 1–2 h post-dosing (6). Pyrazinamide is primarily metabolized to pyrazinoic acid by liver amidase activity, whereas isoniazid is predominantly eliminated by the polymorphic N-acetyltransferase 2 (NAT2) enzyme of the liver and small intestine. The parent drugs are hydrophilic with small molecular weights and low plasma protein binding, facilitating their wide distribution across various tissues and body fluids (5, 7).

Descriptive studies involving TBM patients have reported good pyrazinamide and isoniazid penetration into the CSF, with concentrations approximating those in plasma (7–10). However, there is limited information on the penetration of these drugs into the CNS among patients with HIV-associated TBM, where HIV-induced dysregulation and inflammation of the BBB could affect drug penetration into the CNS (11, 12).

Pyrazinamide and isoniazid are co-administered with rifampicin, a potent enzymatic inducer, and modulator of drug transporter activity, including at the BBB (13). As a result, rifampicin could influence the penetration of these drugs into the CNS, especially at the higher doses currently being evaluated in TBM trials (14, 15).

We therefore characterized the plasma and CSF pharmacokinetics of pyrazinamide and isoniazid among adults with HIV-associated TBM. Additionally, we investigated the influence of high-dose rifampicin (35 mg/kg) and other covariates on the concentrations of both drugs in plasma and CSF.

## MATERIALS AND METHODS

### Study design

This pharmacokinetic study was nested within an open-label, randomized, multi-arm phase 2A trial (LASER-TBM) to assess the safety of high-dose rifampicin, linezolid, and high-dose aspirin for adults with HIV-associated TBM (16). Participants, recruited from four public hospitals in Cape Town and Gqeberha, South Africa, were randomly assigned to one of the three treatment arms within 5 days of starting TB treatment. The control arm received the standard of care for TBM according to WHO weight bands (rifampicin 10 mg/kg, isoniazid 5 mg/kg, pyrazinamide 25 mg/kg, and ethambutol 15 mg/kg), administered as an oral fixed-dose combination. Intervention arms were provided with the standard regimen plus additional rifampicin (35 mg/kg in total, using customized weight bands [17]) and 1,200 mg of linezolid once daily for the first 28 days, reduced to 600 mg for the next 28 days, with (Arm 3) or without (Arm 2) daily aspirin 1,000 mg. All participants received adjunctive corticosteroid therapy with dexamethasone. Experimental therapy was administered for 56 days, after which participants continued standard treatment.

### Pharmacokinetic sampling

Intensive and sparse pharmacokinetic sampling was performed on day 3 (±2 days) and day 28 (±2 days) after study enrollment, respectively. Blood samples on day 3 were collected at pre-dose, 0.5, 1, 2, 3, 6, 8–10, and 24 h post-dose and on day 28 at pre-dose, 2, and 4 h post-dose. One lumbar CSF sample was collected during each pharmacokinetic sampling visit, randomized to windows of 1–3, 3–6, 6–10, or 24 h after drug administration. Samples were processed immediately after collection and stored at −80°C until analysis. Total pyrazinamide and isoniazid concentrations were quantified in all collected plasma and CSF samples; free pyrazinamide concentrations were measured in a subset of plasma samples.

Drug quantification was done using validated liquid chromatography-tandem mass spectrometry assay performed in the Division of Clinical Pharmacology, University of Cape Town. The lower limit of quantification (LLOQ) for the pyrazinamide total and free concentration assays was 0.200 mg/L in plasma samples and 0.234 mg/L for total concentration in CSF samples. For the isoniazid assay, the LLOQ was 0.105 mg/L in plasma and 0.059 mg/L in CSF samples. Additional details regarding the assay methods are provided in the supplementary material (S1).

Patient characteristics, including weight, full blood count, and serum chemistry, were obtained, as were CSF albumin, total protein, glucose, and cell count. Genotyping by qPCR was conducted to identify NAT2 single-nucleotide polymorphisms (SNPs) from whole blood samples of consenting participants. Based on the presence of rs1801279 (NAT2*14), rs1801280 (NAT2*5), rs1799930 (NAT2*6), and rs1799931 (NAT2*7), participants were categorized as slow, intermediate, or rapid acetylators (18). NAT2 genotyping was performed at Inqaba Biotec, South Africa, on the commercially available Agena MassARRAY platform.

### Pharmacokinetic modeling

Nonlinear mixed-effects modeling was conducted using NONMEM version 7.5.1 to develop two separate population pharmacokinetic models describing the concentrations of pyrazinamide and isoniazid in both plasma and CSF. The first-order conditional estimation with eta-epsilon interaction algorithm was used throughout the modeling process. Models were developed using a sequential, non-simultaneous approach, first describing plasma concentrations, followed by incorporation of CSF observations.

To describe pyrazinamide and isoniazid plasma pharmacokinetics, one- and two-compartment disposition models were tested with first-order absorption (with or without lag time or chain of transit compartments) and first-order elimination. Since isoniazid is mostly hepatically cleared, a well-stirred liver model was tested to semi-mechanistically describe the effect of first-pass metabolism (19). The typical value of hepatic blood flow (*Q*_h_) was fixed at 90 L/h, which corresponds to a 70 kg male individual, and the unbound fraction of isoniazid was fixed at 95% (20). The parameters were estimated relative to the pre-hepatic bioavailability, with the typical value of this parameter fixed to 1.

CSF concentrations were described using a hypothetical effect compartment linked to the central (plasma) compartment (21). This method assumes negligible mass transfer from the central to the effect compartment and estimates the plasma-to-CSF equilibrium half-life (HL_Plasma-CSF_) and the CSF-to-plasma pseudo-partition coefficient (PPC_CSF-Plasma_). These parameters describe the delay in the concentration equilibration between the two compartments and the relative drug exposure in CSF compared to plasma at steady state, respectively. Further details on the implementation of the effect compartment for CSF concentration modeling can be found in the supplementary materials (S2).

We tested the effect of body size on disposition parameters using allometric scaling based on weight or fat-free mass (FFM; estimated with the formula by Janmahasatian et al. [22]) and the exponents for clearance and volume were fixed to 0.75 and 1, respectively (23). The effect of NAT2 acetylator phenotype on isoniazid clearance was included early in the model development process, as NAT2 status is known to strongly influence the pharmacokinetics of isoniazid (24). For participants with missing genotypic data, we imputed NAT2 phenotype using a mixture model with the probabilities of slow, intermediate, and rapid NAT2 acetylator fixed to the frequency observed in the individuals with available genotypic data (25). The effect of other covariates including creatinine clearance (calculated using the Cockcroft-Gault formula [26]), study visit, treatment arm, and duration of concomitant rifampicin treatment was tested on plasma pharmacokinetic parameters. Similarly, the effect of CSF albumin, glucose, total protein, polymorphonuclear cells, and the Glasgow Coma Scale was tested on the CSF pharmacokinetic parameters. Details regarding missing covariate imputation can be found in the supplementary materials (S3).

The likelihood ratio test for the drop in objective function (OFV) was used to compare nested models, assumed to be approximately *χ*^2^ distributed with *n* degrees of freedom, where *n* is the number of additional estimated parameters. A supervised stepwise approach was used for model development; a *P* value of 0.05 (ΔOFV ≥ 3.84, df = 1) was used for inclusion of an additional parameter, while a *P* value of 0.01 (ΔOFV ≥ 6.63, df = 1) was required for its retention during backward elimination. Beyond statistical significance, decisions in model development were informed by diagnostic plots, including visual predictive checks (VPCs), as well as physiological plausibility and clinical relevance.

Random effects were included on the pharmacokinetic parameters if statistically significant, using a log-normal distribution (27). Between-subject variability (BSV) was explored for disposition parameters, and between-occasion variability (BOV) was explored for bioavailability and absorption parameters, with an occasion defined as a dosing event and its subsequent observations. Additionally, between-visit variability (BVV) was tested on disposition parameters to evaluate possible differences between visits on day 3 and day 28.

The residual unexplained variability (RUV) was described using an error model with both additive and proportional components, with the additive component constrained to be at least 20% of the LLOQ. Concentrations below the limit of quantification (BLQ) were handled using an adaptation of the M6 method proposed by Beal et al. (28). BLQ concentrations were imputed as half of the drug-matrix-specific LLOQ, and the additive component of the RUV was increased by 50% of the respective LLOQ for these concentrations. In cases of consecutive BLQ concentrations, only one was used: the last one during the absorption phase and/or the first one during the elimination phase. Additional BLQ concentrations were excluded from parameter estimation but retained for simulation-based diagnostics. The precision of the parameter estimates in the final model, indicated by 95% confidence intervals, was evaluated through the sampling importance resampling (SIR) method (29).

The final models were used to calculate individual area under the concentration-time curve from 0 to 24 h post-dose (AUC_0–24h_) and maximum concentration (*C*_max_) values for both drugs in plasma and CSF.

For pyrazinamide plasma samples in which both total and free concentrations were measured, Deming regression was used to estimate the unbound plasma fraction by regressing free against total concentrations with an intercept of 0 (30). The unbound fraction was estimated from the slope of the regression line.

### Simulations

The final parameter estimates were used to simulate steady-state plasma and CSF concentration-time profiles for the typical individual in the cohort following standard doses of pyrazinamide (25 mg/kg) and isoniazid (5 mg/kg).

## RESULTS

### Study data

A total of 414 plasma and 44 CSF concentrations were available for pyrazinamide and isoniazid from 49 individuals during the first visit (day 3) and 34 individuals during the second visit (day 28). For pyrazinamide, 0.97% (4/414, LLOQ: 0.200 mg/L) of plasma observations and 2.3% (1/44, LLOQ: 0.234 mg/L) of CSF observations were BLQ. For isoniazid, 27.5% (114/414, LLOQ: 0.105 mg/L) of plasma observations and 2.3% (1/44, LLOQ: 0.059 mg/L) of CSF observations were BLQ. Thirty-one percent (*n* = 15) of participants were on antiretroviral therapy (ART), which was either efavirenz-based (*n* = 10) or lopinavir/ritonavir-based (*n* = 5). The median time on rifampicin therapy since treatment initiation was 4 days (range: 0–7) for the visit at day 3, and 30 days (range: 26–38) for the second visit on day 28. NAT2 genotypic information was available for 63% (*n* = 31) of the participants: 19% (*n* = 6) were slow, 55% (*n* = 17) as intermediate, and 26% (*n* = 8) as rapid acetylators. For participants with missing NAT2 genotypic data, the mixture model assigned 28% (*n* = 5) as slow acetylators, 56% (*n* = 10) as intermediate acetylators, and 16% (*n* = 3) as rapid acetylators. Baseline characteristics and the NAT2 phenotype distribution are summarized in Table 1.

**TABLE 1:** Participant demographic and clinical characteristics^*a*^

	First visit (day 3)	Second visit (day 28)
	(*n* = 49)	(*n* = 34)
Males	27 (55)	20 (59)
Female	22 (45)	14 (41)
Weight (kg)	60.0 (30.0–107)	62.0 (37.0–105)
Height (cm)^*b*^^,^^*d*^	160 (148–180) [29]	160 (149–180) [19]
Fat-free mass (kg)^*c*^^,^^*d*^	45 (30–59)	45 (32–60)
Age (years)	39 (25–78)	39 (25–57)
Days on rifampicin^*e*^	4 (0–7)	30 (26–38)
CSF total protein (g/L)	1.16 (0.20–55.0) [17]	1.21 (0.20–55.0) [8]
CSF albumin (mg/L)	387 (46.0–7,600) [23]	373 (46.0–1,270) [13]
CSF glucose (mmol/L)	3.05 (0.050–5.90) [17]	2.8 (0.30–5.90) [8]
NAT2 acetylator phenotype		
Slow	6 (12.2)	3 (8.8)
Intermediate	17 (34.7)	13 (38.2)
Rapid	8 (16.3)	6 (17.6)
Missing information	18 (36.8)	12 (35.4)
Antiretroviral therapy (ART)		
On ART	15 (30.6)	10 (29.5)
Efavirenz-based regimen	10 (20.4)	7 (20.5)
Lopinavir/ritonavir-based regimen	5 (10.2)	3 (8.8)
ART Naïve	20 (40.8)	14 (41.1)
Previous ART	14 (28.6)	10 (29.4)

^*a*^ Data are presented as median (range: min–max) or *n* (%). Numbers within brackets indicate the count of IDs with missing values.

^*b*^ The missing heights were estimated using the provided details in the supplementary file, taking into account the sex and weight.

^*c*^ Fat-free mass was calculated by applying the formula from Janmahasatian et al. (22).

^*d*^ The reported median values along with the range (min–max) pertain exclusively to the non-missing data; the imputed values were not included in these calculations.

^*e*^ The total number of days mentioned corresponds to the duration since the initiation of treatment, which commenced approximately 1–3 days before the start date for this study. It is essential to note that all participants were assumed to be on a standard-dose (10 mg/kg) of rifampicin at the commencement of treatment and prior to study enrollment.

### Pharmacokinetic modeling

#### Pyrazinamide

The plasma pharmacokinetics of pyrazinamide was best described by a one-compartment model with first-order absorption and elimination. Implementing absorption delay using transit compartments significantly improved model fit (ΔOFV = –25.52) compared to no delay and provided a better approach than using lag time (ΔOFV = –17.93). Allometric scaling using FFM better captured the influence of body size on drug disposition parameters (ΔOFV = −15.53) than total body weight (ΔOFV = −4.47). Pyrazinamide clearance and volume of distribution for the typical individual in the cohort (FFM: 45 kg) were estimated to be 4.19 L/h and 45.0 L, respectively.

Adding study visits as a covariate improved the model fit (ΔOFV = −120.12) and revealed a 30% (95% confidence interval: 23.8–37.3) increase in pyrazinamide clearance at the second visit. Other covariates, including age, ART, and creatinine clearance, were tested but did not significantly influence the pharmacokinetics of pyrazinamide. In addition, no significant differences were observed between the experimental arms receiving high-dose rifampicin and the control standard of care arm. Similarly, no significant differences were observed between the arm receiving aspirin and the rest of the cohort. Pre-dose concentrations exhibited more variability compared to post-dose concentrations, possibly due to uncertain information on the previous unobserved doses. To address this, the BOV for all absorption parameters and bioavailability was inflated for pre-dose concentrations by a factor estimated to be 2.51. This inclusion notably improved the model fit (ΔOFV = −52.32).

Pyrazinamide CSF concentrations were linked to plasma concentrations with a HL_Plasma-CSF_ of 0.66 hours and a PPC_CSF-Plasma_ of 1.05. No statistically significant effect of CSF albumin, CSF glucose, CSF total protein, CSF polymorphonuclear cells, study arms, or Glasgow Coma Scale was observed on the CSF pharmacokinetic parameters.

A schematic representation of the final model is included in the supplementary materials (Fig. S4), along with a VPC of the pyrazinamide plasma data stratified by study visit and the CSF observations (Fig. S5). The final pharmacokinetic parameters and the respective 95% confidence intervals are shown in Table 2.

**TABLE 2:** Final pharmacokinetic parameters estimate for pyrazinamide and isoniazid

	Typical values (95% confidence interval [CI])^*a*^^,^^*f*^	
Parameter (units)	Pyrazinamide	Isoniazid
Clearance (L/h)^*b*^	4.19 (3.86–4.45)	
Slow acetylator (L/h)^*b*^		14.6 (12.1–17.1)
Intermediate acetylator (L/h)^*b*^		32.2 (28.6–37.0)
Rapid acetylator (L/h)^*b*^		64.7 (54.1–77.7)
Central volume of distribution (L)^*b*^	45.0 (43.4–46.6)	43.6 (39.7–48.7)
Intercompartmental clearance (L/h)^*b*^		5.02 (3.41–6.56)
Peripheral volume of distribution (L)^*b*^		22.3 (15.5–30.3)
Bioavailability (.)	1 Fixed	1 Fixed
Mean absorption transit time (h)	0.291 (0.184–0.380)	0.249 (0.162–0.328)
Number of absorption transit compartments (.)^*d*^	4.25 (3.76–4.88)	5 Fixed
First-order absorption rate constant (h^−1^)	2.5 (2.29–2.68)	2.21 (1.59–3.08)
Hepatic blood flow (L/h)^*b*^^,^^*e*^		76.3 Fixed
BOV scaling factor for unobserved dose (fold change)	2.51 (1.98–3.31)	
Change in clearance on day 28 (%)	+30.2 (+23.8 to +37.3)	
Between-subject variability in clearance (%)	18.5 (15.0–23.0)	25.2 (17.0–30.5)
Between occasion variability in bioavailability (%)	15.8 (11.4–19.2)	32.1 (24.9–39.5)
Between occasion variability in mean absorption transit time (%)	102 (73.5–131)	139 (114–178)
Between occasion variability in absorption rate constant (%)	87.3 (70.6–103)	87.0 (64.6–121)
Proportional error for plasma (%)	8.33 (7.25–9.08)	16.4 (14.5–19.2)
Additive error for plasma (mg/L)^*c*^	0.04 Fixed	0.02 Fixed
CSF-to-plasma pseudo-partition coefficient, PPC_CSF-Plasma_ (.)	1.05 (0.99–1.09)	1.04 (0.76–1.38)
Plasma-to-CSF equilibrium half-life, HL_Plasma-CSF_ (h)	0.66 (0.43–0.90)	3.87 (2.47–7.59)
Proportional error for CSF (%)	11.4 (7.54–16.3)	58.8 (46.9–82.7)
Additive error for CSF (mg/L)^*c*^	0.04 Fixed	0.01 Fixed

^*a*^ Values in parentheses represent the 95% CI, computed using sampling importance resampling (SIR) on the final model.

^*b*^ All the disposition parameters were allometrically scaled. The reported typical values refer to the typical individual in the cohort with fat-free mass of 45 kg.

^*c*^ The estimate of the additive component of the residual unexplained variability did not significantly differ from its lower boundary of 20% of LLOQ, it was consequently fixed to this value.

^*d*^ The number of transit compartments was fixed at 5, based on the previously estimated value, to improve model stability. A sensitivity analysis indicated that this parameter was not critical to the model performance.

^*e*^ The hepatic blood flow value for the typical individual in the cohort (with a fat-free mass of 45 kg) is equivalent to 90 L/h in a male individual with a total body weight of 70 kg and a fat-free mass of 56 kg.

^*f*^ Blank fields indicate parameters not included in the corresponding pharmacokinetic model**.**

**Fig 1:**
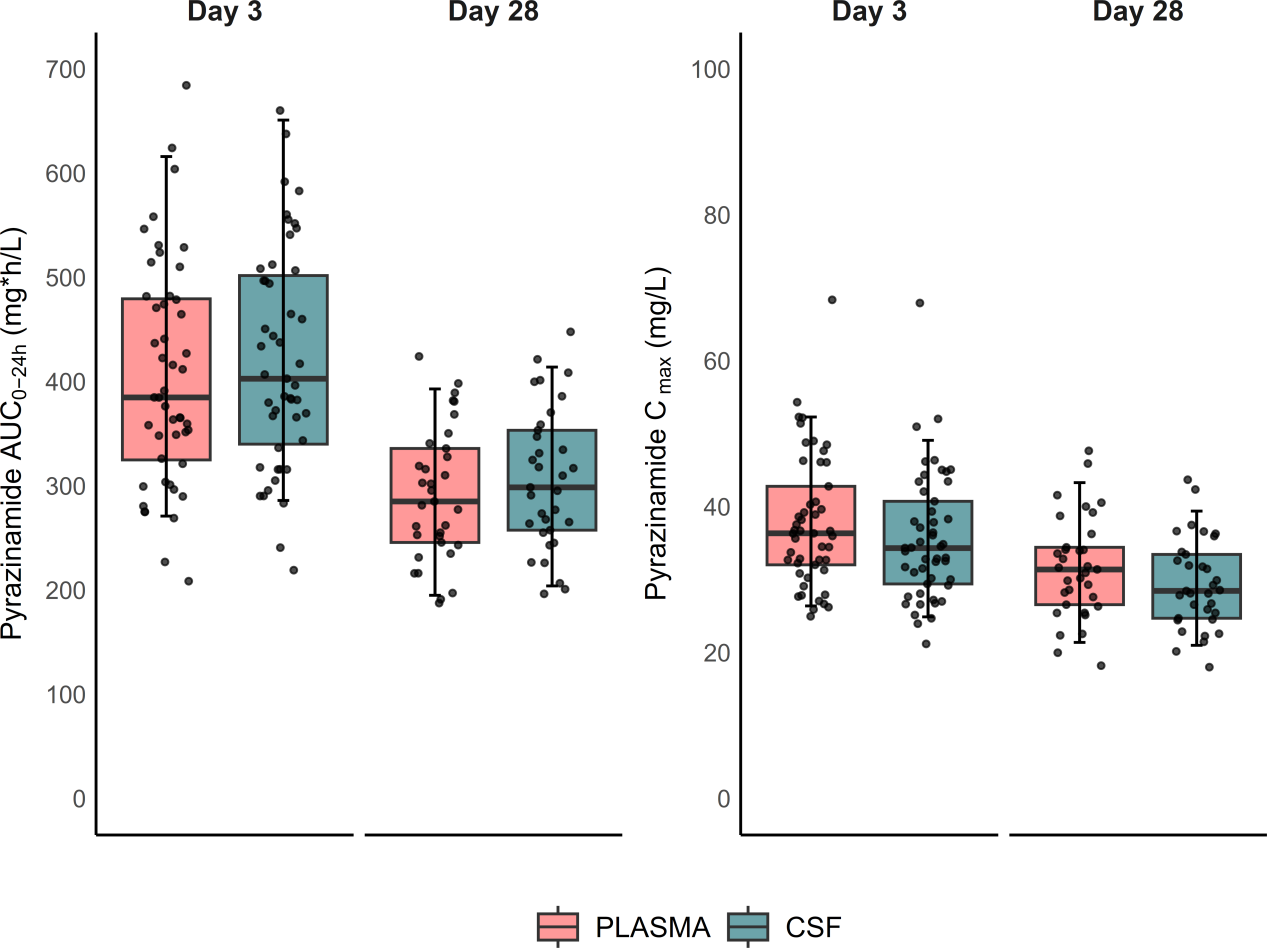
Box-and-whisker plots showing the secondary pyrazinamide model-derived exposure parameters, area under the curve (AUC_0–24h_) and maximum concentration (*C*_max_), stratified by study visit. The whiskers represent the 5th and 95th percentiles of the data.

Model-derived individual AUC_0–24h_ and *C*_max_ values for pyrazinamide are depicted in Fig. 1. Pyrazinamide exposure in plasma and CSF was observed to decrease between the first visit on day 3 and the second visit on day 28.

The unbound plasma fraction of pyrazinamide was 93.3%. Details on the plasma protein binding analysis of pyrazinamide are available in the supplementary materials (Fig. S6).

#### Isoniazid

A two-compartment disposition model better described isoniazid plasma pharmacokinetics compared to a one-compartment model (ΔOFV = –90.42). The implementation of absorption delay using transit compartments significantly improved model fit (ΔOFV = –20.01) compared to no delay and provided a better approach than using lag time (ΔOFV = –12.64). The inclusion of the NAT2 acetylator phenotype on clearance was significant (ΔOFV = −95.78), leading to a drop in the BSV associated with this parameter from 47.7% to 25.2%. Likewise, the inclusion of elimination via hepatic extraction (and first-pass effect) improved the model fit over simple first-order elimination from the central compartment (ΔOFV = −16.20). The inclusion of allometric scaling using FFM on all disposition parameters, including intrinsic clearance and *Q*_h_ (related to the hepatic extraction), yielded better model fit (ΔOFV = −16.95) compared to using body weight (ΔOFV = −9.21).

In a typical participant (FFM: 45 kg), clearance was estimated at 14.6, 32.2, and 64.7 L/h for slow, intermediate, and rapid NAT2 acetylators, respectively.

No statistically significant effect was observed for other covariates, including creatinine clearance, age, ART, concomitant administration of standard versus high-dose rifampicin, and study visits on the different pharmacokinetic parameters of isoniazid. Similarly, no significant differences were observed between the arm receiving aspirin and the rest of the cohort.

Isoniazid HL_Plasma-CSF_ and the PPC_CSF-Plasma_ were 3.87 h and 1.04, respectively. The model did not identify a statistically significant effect of CSF albumin, CSF glucose, CSF total protein, CSF polymorphonuclear cells, study arms, or Glasgow Coma Scale on the isoniazid CSF pharmacokinetic parameters. An illustration of the final model can be found in the supplementary materials (Fig. S7). Table 2 shows parameter estimates of the final pharmacokinetic model and their respective 95% confidence intervals. A VPC for isoniazid plasma data, stratified by NAT2 acetylator phenotype, and for CSF concentrations can be found in the supplementary materials (Fig. S8).

**Fig 2:**
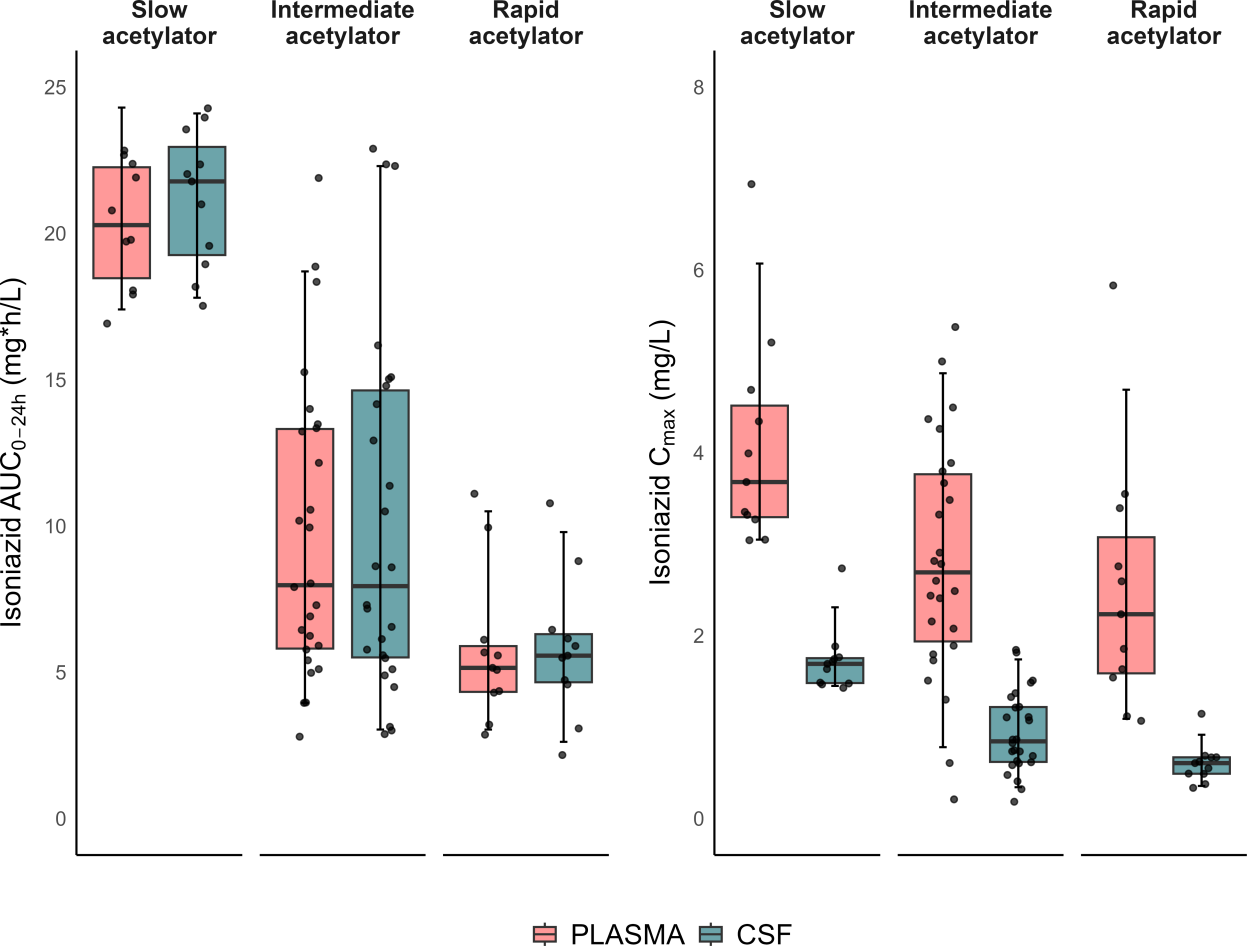
Box-and-whisker plots showing the secondary isoniazid model-derived exposure parameters, area under the curve (AUC_0–24h_) and maximum concentration (*C*_max_), stratified by NAT2 acetylator phenotype. The whiskers represent the 5th and 95th percentiles of the data.

Isoniazid exposure in plasma and CSF was found to differ significantly across the NAT2 acetylator groups. However, no significant differences in isoniazid exposure in plasma or CSF were observed between the two visits for any NAT2 acetylator phenotype group. Figure 2 shows individual AUC_0–24h_ and *C*_max_ values of isoniazid in plasma and CSF from the first visit, stratified by NAT2 acetylator phenotype.

### Simulations

**Fig 3:**
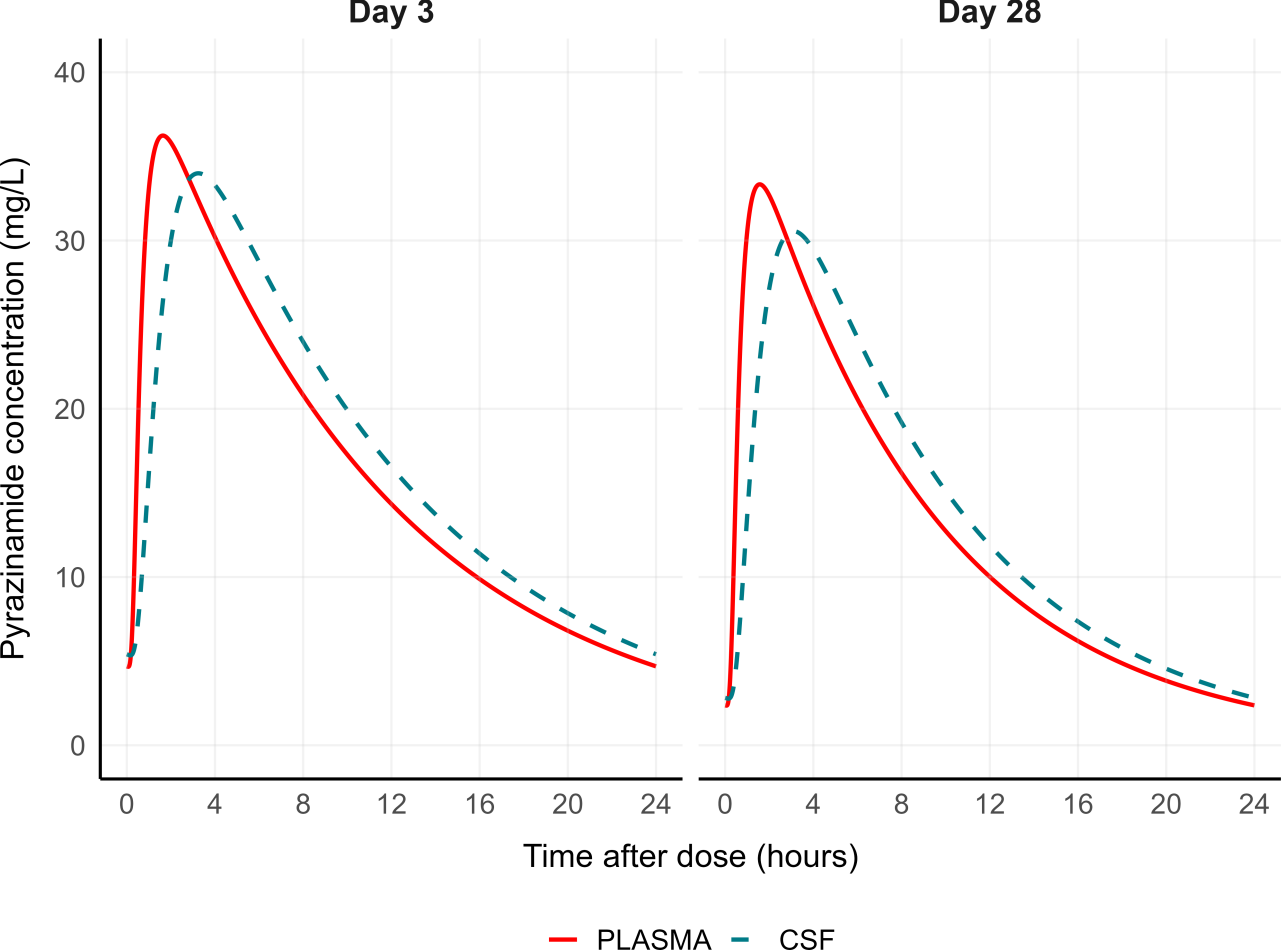
Pyrazinamide simulated steady-state concentration-time profiles in plasma (red solid line) and cerebrospinal fluid (CSF, blue dashed line) for the typical individual in the cohort (fat-free mass of 45 kg) on the first visit (day 3) and the second visit (day 28).

**Fig 4:**
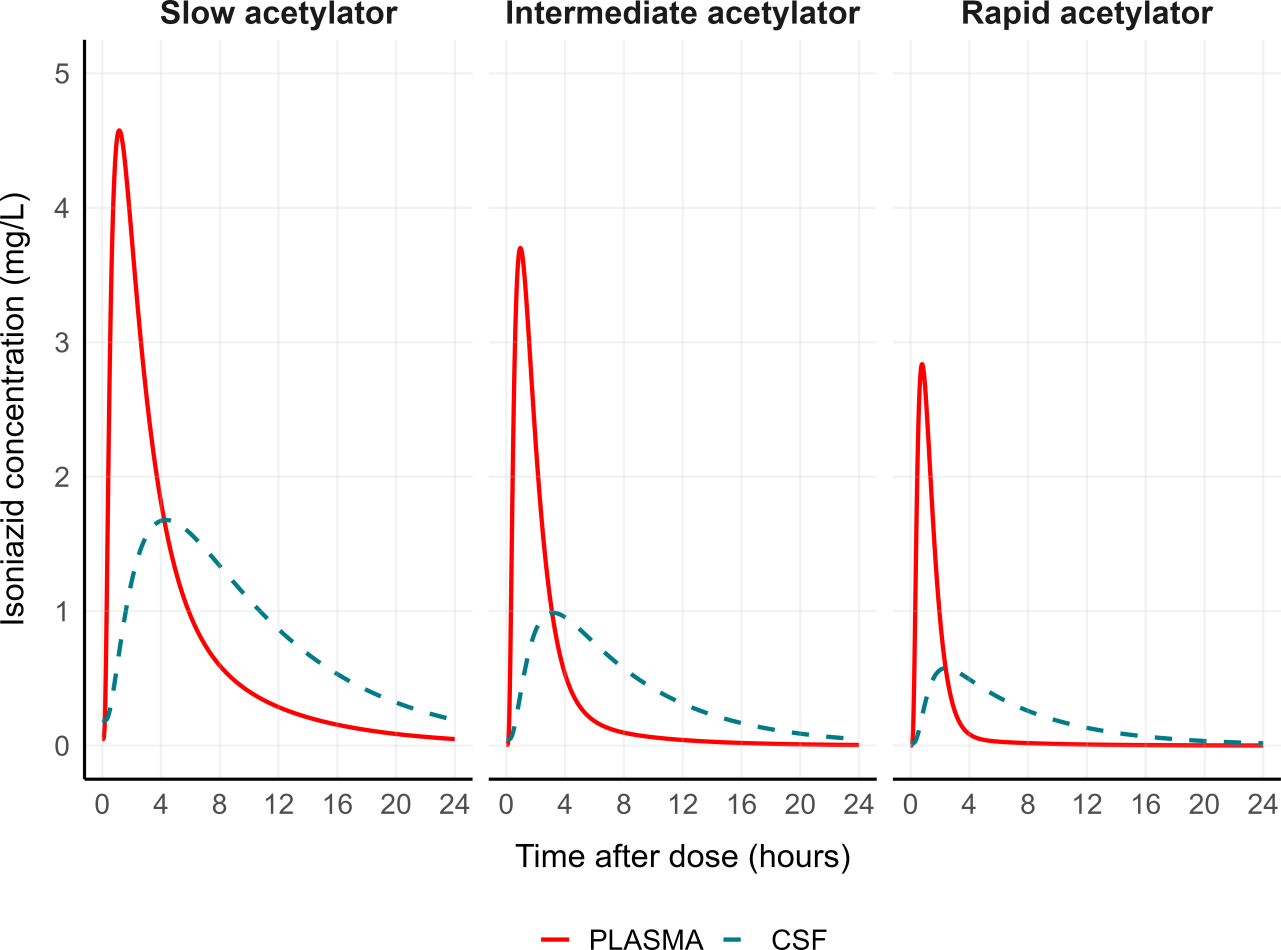
Isoniazid simulated steady-state concentration-time profiles in plasma (red solid line) and cerebrospinal fluid (CSF, blue dashed line) for the typical individual in each NAT2 acetylator phenotype (fat-free mass of 45 kg).

Simulated steady-state plasma and CSF concentration-time profiles for the typical individual in the cohort (FFM: 45 kg) at days 3 and 28 following standard doses of pyrazinamide are depicted in Fig. 3. Likewise, Fig. 4 illustrates simulated steady-state profiles of isoniazid for the typical individual in each NAT2 acetylator phenotype.

## DISCUSSION

We characterized the pharmacokinetics of pyrazinamide and isoniazid in plasma and CSF among South African adults with TBM and HIV co-infection. The penetration of both pyrazinamide and isoniazid into the CSF was excellent, matching their respective plasma exposures (PPC_CSF-Plasma_ ~1). Increasing the rifampicin dose from the standard to 35 mg/kg did not affect the plasma or CSF pharmacokinetics of pyrazinamide or isoniazid. As expected, we observed multi-modal isoniazid clearance governed by the participants’ NAT2 acetylator phenotype. The plasma pharmacokinetics of pyrazinamide and isoniazid in TBM patients from our study aligns with previous reports from pulmonary TB, including the observed 30% increase in pyrazinamide clearance after a median treatment duration of 30 days (31, 32).

Lipophilic drugs can effectively permeate the BBB via lipid-mediated free diffusion, provided the drug has a molecular weight of less than 400 g/mol and forms fewer than eight hydrogen bonds (33). Pyrazinamide and isoniazid are water-soluble molecules, but several factors may explain the extensive CSF distribution observed in our study. Both drugs have low molecular weights of 123.11 and 137.14 g/mol, respectively, enabling them to pass through the BBB paracellularly, unlike the transcellular passage typical of lipophilic agents (34). In the presence of an intact BBB, only the unbound plasma fraction can freely penetrate. We demonstrated that pyrazinamide has a high unbound plasma fraction (93.3%) across the observed concentration range, as does isoniazid with a previously reported unbound fraction of 86% (20). These properties allow both drugs to distribute into various tissues and body fluids, including the CSF. In addition, isoniazid is a substrate for organic anion transporters OAT1 and OAT3, which are expressed in the BBB (35) and may facilitate uptake of isoniazid from the bloodstream into the CSF (36, 37). When adjusted for their unbound plasma fractions, the PPC_CSF-Plasma_ values for pyrazinamide and isoniazid increase to 1.12 and 1.20, respectively, indicating accumulation in the CSF.

The extensive pyrazinamide and isoniazid CSF penetration observed in our study is consistent with previous findings. Patients from an Indonesian TBM cohort (*n* = 52) had pyrazinamide PPC_CSF-Plasma_ values of 0.9 (10), and in a case report of a Chinese patient with drug-resistant TBM, the PPC_CSF-Plasma_ was 0.96 (38). There was no significant difference between the pyrazinamide AUC_0–24h_ in plasma and CSF (379.1 vs 378.6 mg‧h/L, respectively) among Vietnamese adults (*n* = 233) with TBM (39). In the same study, the estimated PPC_CSF-Plasma_ for isoniazid was 0.95 (39), and a cohort study of Korean TBM patients (*n* = 11) found an isoniazid PPC_CSF-Plasma_ of 1.17 (40).

Our simulated typical concentration-time curves (Fig. 3) suggest that the current WHO-recommended dose for pyrazinamide (20–25 mg/kg) is unlikely to achieve CSF concentrations above the critical concentration of 100 mg/L (41). In contrast, the standard isoniazid dose of 5 mg/kg is expected to result in CSF concentrations above the critical concentration of 0.2 mg/L (42), regardless of the NAT2 acetylator phenotype (Fig. 4). These findings should be interpreted with caution, given that pharmacokinetic efficacy targets for pyrazinamide and isoniazid are not established for TBM, and the relative free fraction—the active form—of both drugs in the CSF is unknown.

Median values for pyrazinamide AUC_0–24h_ and *C*_max_ among TBM patients in our study did not differ from those reported in various populations of pulmonary TB patients, including South African, European, and North American cohorts (Fig. 1) (43–46). Also, in line with previous observations among diverse cohorts of patients with pulmonary TB, we found an increase in pyrazinamide clearance during treatment (32). The causes of this phenomenon are not entirely clear, but a potential explanation could be the induction of drug-metabolizing enzymes by rifampicin. The specific mechanism underlying this drug-drug interaction is unknown. One possibility is that rifampicin might induce microsomal deamidase and xanthine oxidase enzymes (47), responsible for pyrazinamide metabolism in the liver, potentially resulting in increased clearance. Enzymatic induction by rifampicin typically manifests within a few days to two weeks of treatment initiation (48), offering a plausible explanation for the decrease in pyrazinamide exposure observed after the second week of treatment. Another possibility is that impaired drug-metabolizing capacity from TB disease (49) is reversed during treatment, resulting in increased pyrazinamide clearance.

Our analysis, consistent with prior studies, identified the NAT2 acetylator phenotype as the major predictor of variability in isoniazid clearance (6, 8, 31, 50). The NAT2 gene is primarily expressed in the intestine and liver, where it influences phase II metabolism of xenobiotics through acetylation mechanisms. The diversity in NAT2 activity can be attributed to different alleles or haplotypes resulting from SNPs. The allele distribution of NAT2 in our study participants aligns with previous findings in a South African population, showing a predominant proportion of intermediate acetylators (51).

The isoniazid exposure observed in this study aligns with previous reports from cohorts of pulmonary TB patients (Fig. 2) (6, 24, 31). The typical clearance value of isoniazid for rapid acetylators (64.7 L/h) in our study was 2.0-fold and 4.4-fold higher than that of intermediate and slow acetylators, respectively, resulting in significant differences in isoniazid exposure across the three NAT2 acetylator groups. However, isoniazid exposure did not differ within any NAT2 acetylator phenotype group between the two study visits.

Our study had some limitations. CSF sampling was limited due to the invasive nature of the lumbar puncture. This was addressed by randomizing CSF sample collection times to various windows across the full dosing period, thus allowing estimation of both the pseudo-partition coefficient and the equilibration delay between plasma and CSF concentrations. Only sparse plasma sampling was available during the second visit, which reduced the sample size for investigating clearance changes. Finally, NAT2 genotype data were not available for all participants in the cohort (because of the requirement for specific consent from unconscious patients), but we implemented a mixture model to assign a phenotype group to individuals with missing information.

### Conclusions

We developed population pharmacokinetic models that adequately describe pyrazinamide and isoniazid pharmacokinetics in plasma and CSF from adults with HIV-associated TBM. Our models showed that the plasma pharmacokinetics of pyrazinamide and isoniazid in TBM closely resemble their behavior in pulmonary TB patients, even when co-administered with high doses of rifampicin, providing evidence for combined use. Moreover, the models showed that the CSF exposure of these drugs mirrors their exposure in plasma. These findings support further efficacy evaluation of pyrazinamide and isoniazid in TBM and provide a tool to explore alternative dosing strategies for these drugs in TBM.

## Data Availability

The data sets supporting the findings of this study are available from the corresponding author, P.D., upon reasonable request.

## SUPPLEMENTAL MATERIAL

Supplemental material aac.00099-25-s0001.docx Sample quantification procedures (S1); additional modeling details (S2 and S3); Supplemental figures (S4 to S8); the NONMEM code used for the final pharmacokinetic models (S9 and S10).

ASM does not own the copyrights to Supplemental Material that may be linked to, or accessed through, an article. The authors have granted ASM a non-exclusive, world-wide license to publish the Supplemental Material files. Please contact the corresponding author directly for reuse.
