# Supplementary material for: Population pharmacokinetics of pyrazinamide and isoniazid in plasma and cerebrospinal fluid from South African adults with tuberculous meningitis
Source: Antimicrob Agents Chemother. 2025 Jul 1;69(8):e00099-25. doi: 10.1128/aac.00099-25 (PMC12327010; doi:10.1128/aac.00099-25)
Supplement: Supplemental material — Sample quantification procedures (S1); additional modeling details (S2 and S3); Supplemental figures (S4 to S8); the NONMEM code used for the final pharmacokinetic models (S9 and S10). [file aac.00099-25-s0001.docx]

**SUPPLEMENTARY MATERIAL**

**S1. Sample quantification**

**S1.1 Plasma samples**

Total and free pyrazinamide and total isoniazid plasma concentrations were determined using liquid chromatography-tandem mass spectrometry (LC-MS/MS), performed at the Division of Clinical Pharmacology, University of Cape Town. The calibration range for the total and free pyrazinamide assays extended from 0.200 mg/L (lower limit of quantification [LLOQ]) to 80.0 mg/L. For the total pyrazinamide assay, the inter-day accuracy ranged from 99.7% to 102.3%, with a percent coefficient of variation (%CV) for precision between 0.7% and 2.8%. For the free pyrazinamide assay, the inter-day accuracy ranged from 90.0% to 95.5%, and the %CV for precision was between 4.3% and 10.3%. For the isoniazid assay, the calibration range was from 0.105 mg/L (LLOQ) to 25.0 mg/L, with inter-day accuracy varying between 99.1% and 101.5%, and a %CV for precision ranging from 2.6% to 3.2%.

**S1.2. Cerebrospinal fluid (CSF) samples**

Pyrazinamide and isoniazid CSF samples were analysed using a validated LC-MS/MS assay conducted at the Division of Clinical Pharmacology, University of Cape Town. Sample extraction was performed with Strata-X 96-well solid phase extraction columns, followed by liquid chromatographic separation on an Atlantis T3, 100A, 3 µm, 2.1 mm x 100 mm analytical column. The separation utilized a gradient flow with mobile phase A: 0.1% formic acid in water, and mobile phase B: 0.1% formic acid in a mixture of acetonitrile and methanol (50:50, v/v) at a flow rate of 0.25 mL/min. An AB Sciex API 4000 mass spectrometer, operated at unit resolution in multiple reaction monitoring mode, monitored the transitions of the protonated precursor ions at 138.1, 124.1, 142.1, and 128.1 to the product ions 79.2, 97.2, 125.3, and 84.0 for INH, PZA, INH-d4, and PZA-15N,d3, respectively. Calibration curves were fitted using quadratic regressions (weighted by 1/x) based on peak area ratios, covering the ranges of 0.059 - 15.0 mg/L for isoniazid and 0.234 - 60.0 mg/L for pyrazinamide. The combined accuracy (%Nom) and precision statistics of the limit of quantification, low, medium, and high-quality controls (from three validation batches, N=18) for isoniazid and pyrazinamide were between 88.4% and 107.8% for accuracy, and between 2.5% and 10.1% for precision, respectively.

**S2. Effect compartment implementation for CSF concentrations modelling**

The CSF concentrations were modelled as dependent on the plasma concentrations, following the method suggested by Sheiner *et al*. (21). This modelling approach involved implementing a hypothetical effect compartment linked to the central compartment. This method assumes negligible drug transfer from the central to the effect compartment, as well as negligible volume of the effect compartment compared to the central compartment. The following differential equation summarizes the kinetic of the effect compartment:

$$\frac{{dC}_{CSF}}{dt}=k_{Plasma-CSF}\cdot\left( PPC\cdot C_{Plasma}-C_{CSF} \right)$$

The equilibrium between the central compartment (plasma) and the effect compartment (CSF) is governed by the first-order rate constant k_Plasma−CSF_. PPC represents the pseudo-partition coefficient, while C_Plasma_ and C_CSF_ denote the drug concentration in plasma and CSF at time 𝑡, respectively. The k_Plasma−CSF_ was used to calculate the plasma to CSF equilibration half-life (HL_Plasma-CSF_) employing the equation:

$${HL}_{Plasma-CSF}=\frac{ln\left( 2 \right)}{k_{Plasma-CSF}}$$

**S3. Missing covariates imputation**

Missing covariates such as CSF protein, CSF albumin, and CSF glucose levels were imputed by the median. Missing heights were imputed using a methodology previously developed by Johansson and Karlsson (52). First, patients’ characteristics (sex, weight, height) from a similar population were used to develop a multiple linear regression model for heights versus weight by sex and accounting for residual variability in heights. Subsequently, this multiple linear regression model was employed in NONMEM to estimate the missing heights. The imputation model is expressed by the equation:

$$Ht_{i}=\beta+\alpha.Wt_{i}.e^{\eta_{i}}$$

Where Hti represents the individual height in meters, Wti is the individual weight in kilograms, β and α denote the model mean intercept and slope, respectively, and ηi is the random effect capturing the individual deviation from the mean values. The ηi values are assumed to follow a normal distribution with a mean of zero and a variance of ω2. The specific values for β and α are 1.51 and 0.00133 for females, and 1.53 and 0.00281 for males, respectively. The variance values were 0.00215 for females and 0.00170 for males.

**S4.** Schematic representation of the pyrazinamide final model


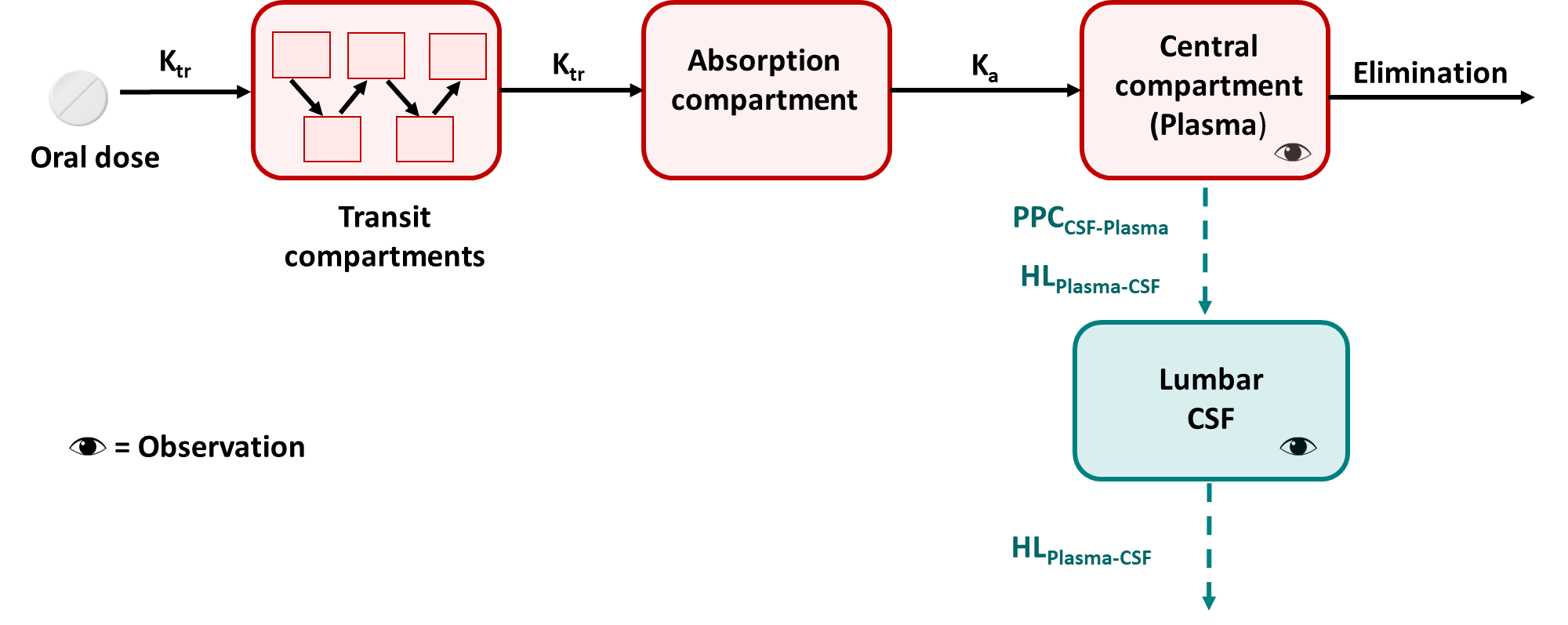


Schematic representation of the final model. K_tr_ is the first-order rate constant for drug passage through transit compartments. HL_Plasma-CSF_ is the equilibration half-life between plasma and cerebrospinal fluid, which describe how soon the change in plasma is reflected in the CSF. PPC_CSF-Plasma_ is the pseudo-partition coefficient which represents the relative drug exposure in CSF compared to plasma at steady state.


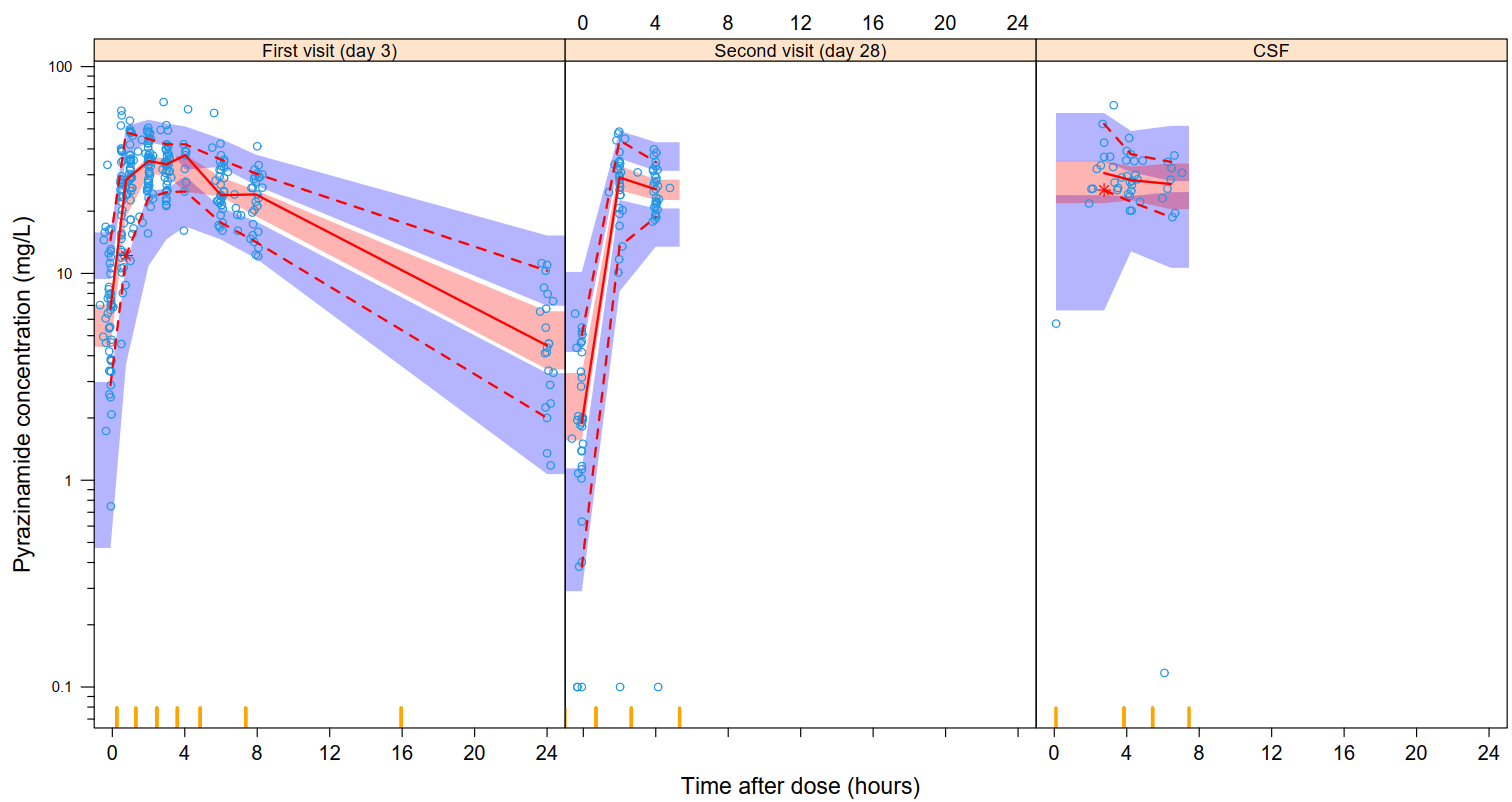
**S5.** Visual predictive check for pyrazinamide plasma (stratified by study visit) and cerebrospinal fluid (CSF) concentrations versus time after dose. The circles represent the original data, the dashed and solid lines are the 10^th^, 50^th^, and 90^th^ percentiles of the observed data, while the shaded areas represent the corresponding model-predicted 95% confidence intervals.


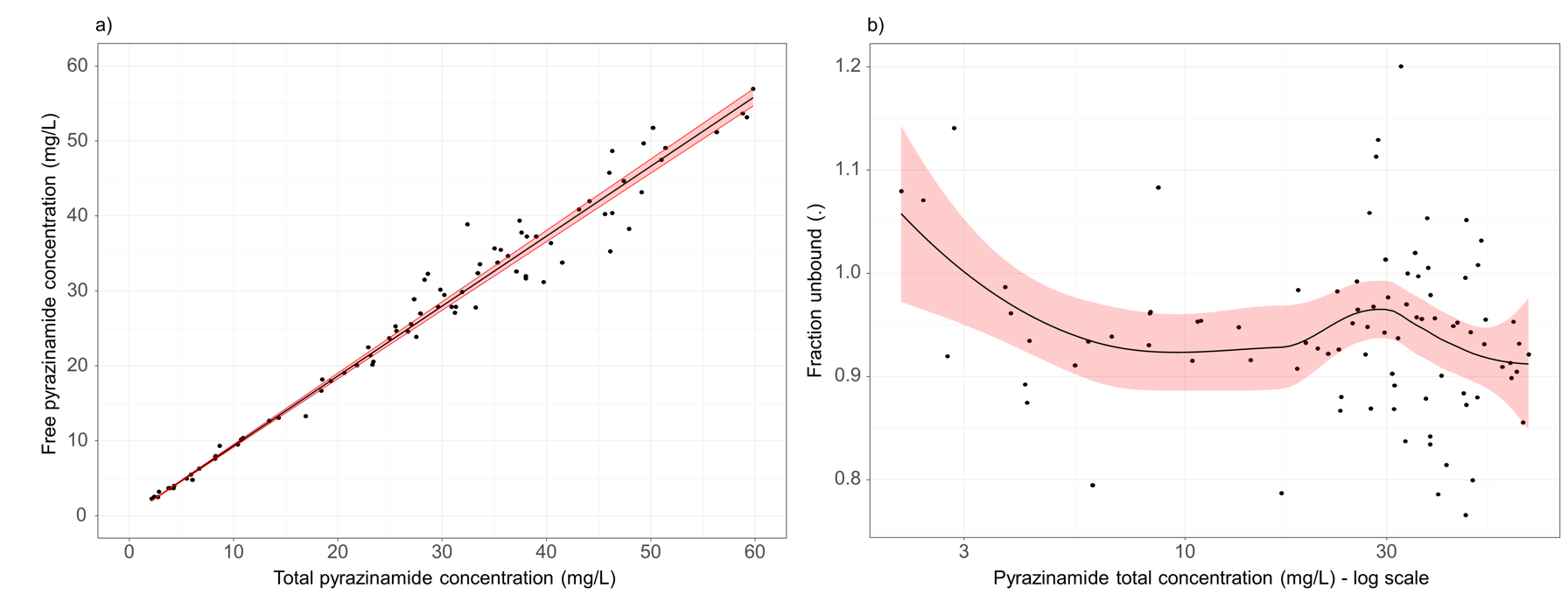


y= 0.933 ‧ x

Pearson’s r = 0.987

**Figure S6:** Binding plots for plasma protein binding of pyrazinamide a) Free versus total concentration (mg/L) in the same sample. The slope of the regression equation represents the unbound fraction and was estimated using generalized Deming regression with constant error assumption (30). b) LOESS regression of fraction unbound versus total pyrazinamide concentrations (mg/L). There was no apparent trend between the two variables.

**S7**. Schematic representation of the isoniazid final model


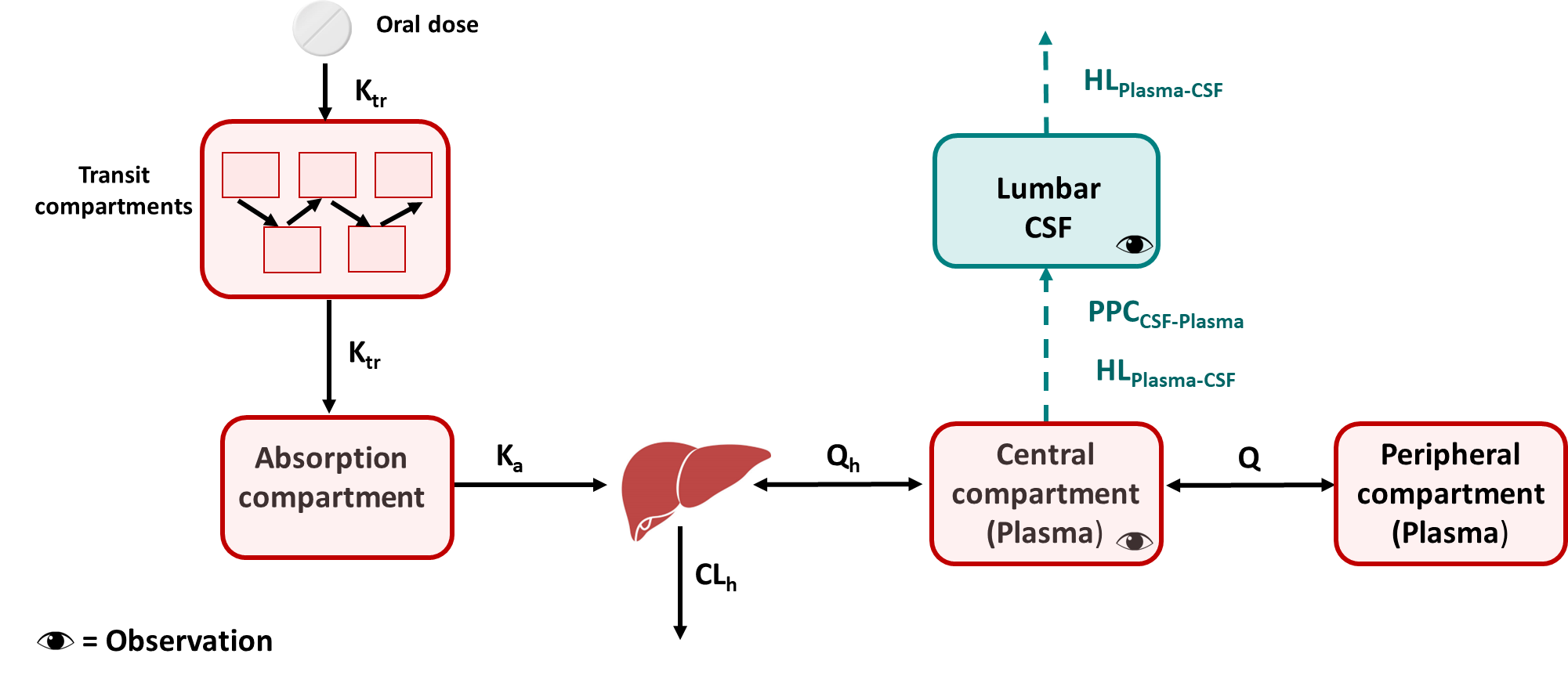


Schematic representation of the final model. K_tr_ is the first-order rate constant for drug passage through transit compartments. Q_h_ is the hepatic blood flow and Q is the intercompartmental clearance. Cl_h_ is the hepatic clearance. HL_Plasma-CSF_ is the equilibration half-life between plasma and cerebrospinal fluid, which describe how soon the change in plasma is reflected in the CSF. PPC_CSF-Plasma_ is the pseudo-partition coefficient which represents the relative drug exposure in CSF compared to plasma at steady state.


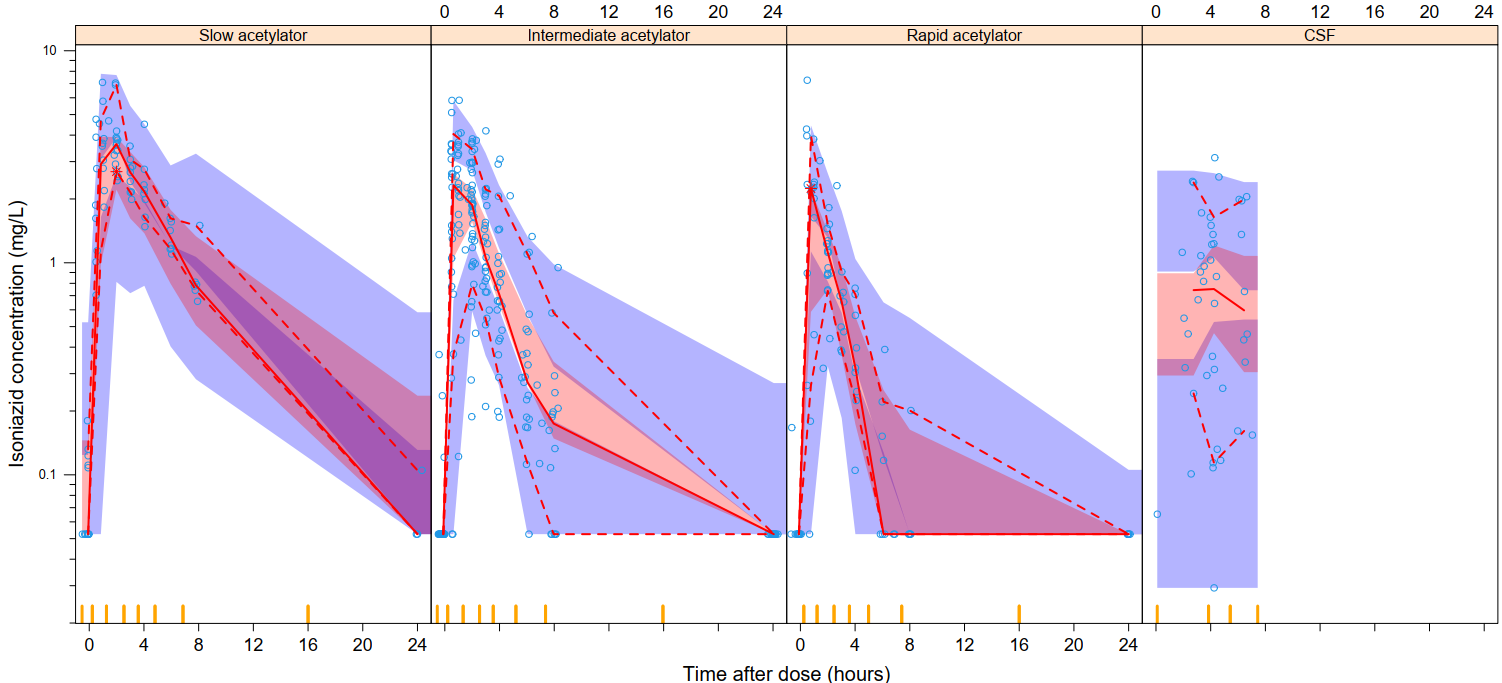


**S8.** Visual predictive check for isoniazid plasma (stratified by NAT2 acetylator phenotype) and cerebrospinal fluid concentrations versus time after dose. The circles represent the original data, the dashed and solid lines are the 10^th^, 50^th^, and 90^th^ percentiles of the observed data, while the shaded areas represent the corresponding model-predicted 95% confidence intervals.

**S9.** Pyrazinamide final model NONMEM code

;----------------------------------------------------------------------------------------------------------------------

; Settings for the memory of NONMEM

$SIZES PD=-1000 LVR=-150 LTH=-200 MAXFCN=10000000 LNP4=-150000

;----------------------------------------------------------------------------------------------------------------------

$PROBLEM -

;----------------------------------------------------------------------------------------------------------------------

$INPUT -

;----------------------------------------------------------------------------------------------------------------------

$DATA -

;----------------------------------------------------------------------------------------------------------------------

$ABBR DERIV2=NO

$SUBROUTINE ADVAN14 TRANS1 TOL=9 ATOL=9

;----------------------------------------------------------------------------------------------------------------------

$MODEL

NCOMPARTMENTS = 3

COMP=(ABS DEFDOSE)

COMP=(CENTRAL)

COMP=(CSF)

;----------------------------------------------------------------------------------------------------------------------

$PK

;Defining between-occasion variability---------------------------------------------------------------------------------

BOVCL = 0

BOVBIO = 0

BOVKA = 0

BOVMTT = 0

;Occasion 1-----------------------------------------------------------------------------------------------------------

IF(OCC==1) THEN

BOVCL = ETA(8)

BOVBIO = ETA(13)

BOVKA = ETA(18)

BOVMTT = ETA(23)

ENDIF

;Occasion 2-----------------------------------------------------------------------------------------------------------

IF (OCC==2) THEN

BOVCL = ETA(9)

BOVBIO = ETA(14)

BOVKA = ETA(19)

BOVMTT = ETA(24)

ENDIF

;Occasion 3-----------------------------------------------------------------------------------------------------------

IF(OCC==3) THEN

BOVCL = ETA(10)

BOVBIO = ETA(15)

BOVKA = ETA(20)

BOVMTT = ETA(25)

ENDIF

;Occasion 4-----------------------------------------------------------------------------------------------------------

IF (OCC==4) THEN

BOVCL = ETA(11)

BOVBIO = ETA(16)

BOVKA = ETA(21)

BOVMTT = ETA(26)

ENDIF

;Occasion 5-----------------------------------------------------------------------------------------------------------

IF (OCC==5) THEN

BOVCL = ETA(12)

BOVBIO = ETA(17)

BOVKA = ETA(22)

BOVMTT = ETA(27)

ENDIF

;----------------------------------------------------------------------------------------------------------------------

; Implementing an extra layer of variability by allowing BOV on absorption-related parameters to differ based on the

; OBSERVED column 0 = unobserved doses and subsequent samples; 1 = doses and samples observed on the PK sampling day)--

IF(OBSERVED.EQ.0) THEN

BOVBIO = BOVBIO*THETA(8)

BOVKA = BOVKA*THETA(8)

BOVMTT = BOVMTT*THETA(8)

ENDIF

IF (OBSERVED.EQ.1) THEN

BOVBIO =BOVBIO

BOVKA = BOVKA

BOVMTT = BOVMTT

ENDIF

;----------------------------------------------------------------------------------------------------------------------

;Defining between-subject variability---------------------------------------------------------------------------------

BSVCL = ETA(1)

BSVV = ETA(2)

BSVKA = ETA(3)

BSVBIO = ETA(4)

BSVMTT = ETA(5)

BSVKE0 = ETA(6)

BSVPPC = ETA(7)

;----------------------------------------------------------------------------------------------------------------------

;Implementing allometric scaling---------------------------------------------------------------------------------------

TVFFM = 45 ; Typical value of fat-free mass in the study cohort

ALLMCL_FFM = (FFM/TVFFM)**0.75 ; Allometric scaling factor for the clearance parameter

ALLMV_FFM = (FFM/TVFFM) ; Allometric scaling factor for the volume of distribution parameter

;Implementing percent change in clearance between study visits--------------------------------------------------------

IF (PK_VISIT.EQ.3) PK_VISIT_CL = 1

IF (PK_VISIT.EQ.28) PK_VISIT_CL = 1 + THETA(7)

;----------------------------------------------------------------------------------------------------------------------

; Defining typical values of the pharmacokinetic parameters----------------------------------------------------------

TVCL = THETA(1)*ALLMCL_FFM*PK_VISIT_CL

TVV = THETA(2)*ALLMV_FFM

TVKA = THETA(3)

TVBIO = THETA(4)

TVMTT = THETA(5)

TVNN = THETA(6)

; Typical values of CSF penetration parameters--------------------------------------------------------------------------

TVKE0 = THETA(11)

TVPPC = THETA(12)

;----------------------------------------------------------------------------------------------------------------------

; Defining parameters--------------------------------------------------------------------------------------------------

CL = TVCL*EXP(BSVCL+BOVCL) ; CLEARANCE

V = TVV*EXP(BSVV) ; CENTRAL VOL.

KA = TVKA*EXP(BSVKA+BOVKA) ; ABS. RATE CONSTANT

BIO = TVBIO*EXP(BSVBIO+BOVBIO) ; BIOAVAILABILITY

MTT = TVMTT*EXP(BSVMTT+BOVMTT) ; MTT TIME

NN = TVNN ; Number of transit compartments

; CSF penetration parameters

KE0 = TVKE0 * EXP(BSVKE0) ; Plasma-to-CSF equilibration first-order rate constant

PPC = TVPPC * EXP(BSVPPC) ; CSF-to-plasma pseudo-partition coefficient

;----------------------------------------------------------------------------------------------------------------------

; re-parameterization

K = CL/V ; First-order elimination rate constant

;----------------------------------------------------------------------------------------------------------------------

; Transit compartment absorption

F1=0

KTR = (NN+1)/MTT

IF (NEWIND/=2.OR.EVID>=3) THEN

TNXD=TIME

PNXD=AMT

TIMEDOSE = TIME

AMOUNTDOSE = AMT

ENDIF

TDOS=TNXD

PD=PNXD

IF(AMT>0) THEN

TNXD=TIME

PNXD=AMT

ENDIF

PIZZA = LOG(BIO*PD*KTR + 1E-12) - GAMLN(NN+1)

;----------------------------------------------------------------------------------------------------------------------

A_0(1) = 1E-12; ABS CMT

A_0(2) = 1E-12; CENTRAL CMT

A_0(3) = 1E-12; CSF CMT

;----------------------------------------------------------------------------------------------------------------------

$DES

C2 = A(2)/V

TEMPO = T-TDOS

KTT = 0

TRANSIT = 0

IF(PD.GT.0.AND.TEMPO.GT.0) THEN

KTT = KTR*(TEMPO)

TRANSIT = EXP(PIZZA+NN*LOG(KTT)-KTT)

ENDIF

DADT(1) = TRANSIT -KA*A(1)

DADT(2) = KA*A(1) -K*A(2)

DADT(3) = KE0*(PPC*C2 - A(3))

;----------------------------------------------------------------------------------------------------------------------

$ERROR

; Error model for plasma observations----------------------------------------------------------------------------------

LLOQ_P = 0.2

CENS_THR_P = LLOQ_P

CP = A(2)/V

IPRED_P = CP

PROP_P = IPRED_P*THETA(9)

ADD_P = THETA(10)+(CENS_THR_P*0.2)

IF (ICALL/=4.AND.CENS==1.AND.DVID==1) THEN

ADD_P = ADD_P +(LLOQ_P*0.5)

ENDIF

IF (ICALL/=4.AND.CENS==2.AND.DVID==1) THEN

PROP_P = 0

ADD_P = 10000000000

NO_FIT = 1

ENDIF

W_P= SQRT((ADD_P)**2+(PROP_P)**2)

; Error model for CSF observations----------------------------------------------------------------------------------

LLOQ_E = 0.234

CENS_THR_E = LLOQ_E

CE = A(3)

IPRED_E = CE

PROP_E = IPRED_E*THETA(13)

ADD_E = THETA(14) + (0.2*CENS_THR_E)

IF(ICALL/=4.AND.CENS==1.AND.DVID==3) THEN

ADD_E = ADD_E + (LLOQ_E*0.5)

ENDIF

W_E = SQRT((ADD_E)**2 + (PROP_E)**2)

ERROR_P = W_P * ERR(1)

ERROR_E = W_E * ERR(1)

; Redefine IPRED, residual error weighting, and error term based on DVID--------------------------------------------

IPRED = IPRED_P

W = W_P

ERROR_TERM = ERROR_P

IF(DVID==3) THEN

IPRED = IPRED_E

W = W_E

ERROR_TERM = ERROR_E

ENDIF

; Protective code--------------------------------------------------------------------------------------------------

IF (W.LE.0.000001) W=0.000001

IRES=DV-IPRED

IWRES=IRES/W

Y = IPRED + ERROR_TERM

; To prevent simulation (ICALL==4) of negative values--------------------------------------------------------------

IF (DVID==1.AND.ICALL==4.AND.Y<=LLOQ_P) Y=LLOQ_P/2

IF (DVID==3.AND.ICALL==4.AND.Y<=LLOQ_E) Y=LLOQ_E/2

; To calculate time after dose-------------------------------------------------------------------------------------

IF(AMT>0) THEN

TIMEDOSE = TIME

AMOUNTDOSE = AMT

ENDIF

TAD = TIME-TIMEDOSE

TSOD = TAD

VARCL = BSVCL + BOVCL

VARBIO = BSVBIO + BOVBIO

VARAUC = BSVBIO + BOVBIO - BSVCL - BOVCL

VARABS = BOVKA + BSVKA -BSVMTT - BOVMTT ;BSVLAG - BOVLAG

; Retrieve amount in each compartment-----------------------------------------------------------------------------

AA1 = A(1)

AA2 = A(2)

AA3 = A(3)

;-------------------------------------------------------------------------------------------------------------------

; Initial estimates ------------------------------------------------------------------------------------------------

$THETA

(4.19) ; 1 CL [L/h]

(45) ; 2 V [L]

(2.5) ; 3 KA [1/h]

(1) FIX ; 4 BIO

(0.294) ; 5 MTT

(4.25) ; 6 NN []

(0.302) ; 7 PK_VISIT_CL [%]

(2.51) ; 8 BOV SCALING PRE-DOSE [Folds]

(0.083) ; 9 PROP [%]

(0) FIX ; 10 ADD [mg/L]

(1.04) ; 11 KE0 [1/h]

(1.05) ; 12 PPC []

(0.114) ; 13 PROP_E [%]

(0) FIX ; 14 ADD_E [mg/L]

;-------------------------------------------------------------------------------------------------------------------

$OMEGA BLOCK(1) 0.0341 ; 1 BSVCL

$OMEGA BLOCK(1) 0 FIX ; 2 BSVV

$OMEGA BLOCK(1) 0 FIX ; 3 BSVKA

$OMEGA BLOCK(1) 0 FIX ; 4 BSVBIO

$OMEGA BLOCK(1) 0 FIX ; 5 BSVMTT

$OMEGA BLOCK(1) 0 FIX ; 6 BSVKE0

$OMEGA BLOCK(1) 0 FIX ; 7 BSVPPC

;-------------------------------------------------------------------------------------------------------------------

$OMEGA BLOCK(1) 0 FIX ; 8 BOVCL

$OMEGA BLOCK(1) SAME ; 9 BOVCL

$OMEGA BLOCK(1) SAME ; 10 BOVCL

$OMEGA BLOCK(1) SAME ; 11 BOVCL

$OMEGA BLOCK(1) SAME ; 12 BOVCL

;-------------------------------------------------------------------------------------------------------------------

$OMEGA BLOCK(1) 0.0249 ; 13 BOVBIO

$OMEGA BLOCK(1) SAME ; 14 BOVBIO

$OMEGA BLOCK(1) SAME ; 15 BOVBIO

$OMEGA BLOCK(1) SAME ; 16 BOVBIO

$OMEGA BLOCK(1) SAME ; 17 BOVBIO

;-------------------------------------------------------------------------------------------------------------------

$OMEGA BLOCK(1) 0.763 ; 18 BOVKA

$OMEGA BLOCK(1) SAME ; 19 BOVKA

$OMEGA BLOCK(1) SAME ; 20 BOVKA

$OMEGA BLOCK(1) SAME ; 21 BOVKA

$OMEGA BLOCK(1) SAME ; 22 BOVKA

;-------------------------------------------------------------------------------------------------------------------

$OMEGA BLOCK(1) 1.04 ; 23 BOVMTT

$OMEGA BLOCK(1) SAME ; 24 BOVMTT

$OMEGA BLOCK(1) SAME ; 25 BOVMTT

$OMEGA BLOCK(1) SAME ; 26 BOVMTT

$OMEGA BLOCK(1) SAME ; 27 BOVMTT

;-------------------------------------------------------------------------------------------------------------------

$SIGMA 1 FIX

;-------------------------------------------------------------------------------------------------------------------

$ESTIMATION MSFO=run.msf MAXEVAL=9999 PRINT=1 METHOD=1 INTER NOABORT

NSIG=3 SIGL=9

NONINFETA=1 ETASTYPE=1

;$COVARIANCE PRINT=E ; MATRIX=S

;-------------------------------------------------------------------------------------------------------------------

$TABLE ...

;-------------------------------------------------------------------------------------------------------------------

**S10.** Isoniazid final model NONMEM code

;----------------------------------------------------------------------------------------------------------------------

; Settings for the memory of NONMEM

$SIZES PD=-1000 LVR=-150 LTH=-200 MAXFCN=10000000 LNP4=-150000

;----------------------------------------------------------------------------------------------------------------------

$PROBLEM -

;----------------------------------------------------------------------------------------------------------------------

$INPUT -

;----------------------------------------------------------------------------------------------------------------------

$DATA -

;----------------------------------------------------------------------------------------------------------------------

$ABBR DERIV2=NO

$SUBROUTINE ADVAN14 TRANS1 TOL=9 ATOL=9

;----------------------------------------------------------------------------------------------------------------------

$MODEL

NCOMPARTMENTS=4

COMP=(ABS DEFDOSE)

COMP=(CENTRAL)

COMP=(PERI1)

COMP=(CSF)

;----------------------------------------------------------------------------------------------------------------------

$PK

;Defining between-occasion variability---------------------------------------------------------------------------------

BOVCL = 0

BOVBIO = 0

BOVKA = 0

BOVMTT = 0

;Occasion 1-----------------------------------------------------------------------------------------------------------

IF(OCC==1) THEN

BOVCL = ETA(10)

BOVBIO = ETA(15)

BOVKA = ETA(20)

BOVMTT = ETA(25)

ENDIF

;Occasion 2-----------------------------------------------------------------------------------------------------------

IF (OCC==2) THEN

BOVCL = ETA(11)

BOVBIO = ETA(16)

BOVKA = ETA(21)

BOVMTT = ETA(26)

ENDIF

;Occasion 3-----------------------------------------------------------------------------------------------------------

IF(OCC==3) THEN

BOVCL = ETA(12)

BOVBIO = ETA(17)

BOVKA = ETA(22)

BOVMTT = ETA(27)

ENDIF

;Occasion 4-----------------------------------------------------------------------------------------------------------

IF (OCC==4) THEN

BOVCL = ETA(13)

BOVBIO = ETA(18)

BOVKA = ETA(23)

BOVMTT = ETA(28)

ENDIF

;Occasion 5-----------------------------------------------------------------------------------------------------------

IF (OCC==5) THEN

BOVCL = ETA(14)

BOVBIO = ETA(19)

BOVKA = ETA(24)

BOVMTT = ETA(29)

ENDIF

;----------------------------------------------------------------------------------------------------------------------

;Defining between-subject variability---------------------------------------------------------------------------------

BSVCL = ETA(1)

BSVV = ETA(2)

BSVKA = ETA(3)

BSVBIO = ETA(4)

BSVV3 = ETA(5)

BSVQ = ETA(6)

BSVMTT = ETA(7)

BSVKE0 = ETA(8)

BSVPPC = ETA(9)

;----------------------------------------------------------------------------------------------------------------------

;Implementing allometric scaling---------------------------------------------------------------------------------------

TVFFM = 45 ; Typical value of fat-free mass in the study cohort

ALLMCL_FFM = (FFM/TVFFM)**0.75 ; Allometric scaling factor for the clearance parameter

ALLMV_FFM = (FFM/TVFFM) ; Allometric scaling factor for the volume of distribution parameter

; Allometry for liver extraction

ALLMCL_FFM_HEP = (FFM/56.1)**0.75

ALLMV_FFM_HEP = (FFM/56.1)

;----------------------------------------------------------------------------------------------------------------------

;----------------------------------------------------------------------------------------------------------------------

; Defining typical values of the pharmacokinetic parameters----------------------------------------------------------

; Clearance stratification based on acetylator phenotype--------------------------------------------------------------

; NAT2_phenotype – Dataset column storing NAT2 phenotype (observed or imputed via mixture model)

; 0 Slow acetylator

; 1 Intermediate acetylator

; 2 Rapid acetylator

IF(NAT2_phenotype.EQ.0) THEN

TVCL = THETA(1)*ALLMCL_FFM

ENDIF

IF (NAT2_phenotype.EQ.1) THEN

TVCL = THETA(2)*ALLMCL_FFM

ENDIF

IF (NAT2_phenotype.EQ.2) THEN

TVCL = THETA(3)*ALLMCL_FFM

ENDIF

TVV = THETA(4)*ALLMV_FFM

TVKA = THETA(5)

TVBIO = THETA(6)

TVV3 = THETA(7)*ALLMV_FFM

TVQ = THETA(8)*ALLMCL_FFM

TVMTT = THETA(9)

TVNN = THETA(10)

; Hepatic extraction---------------------------------------------------------------------------------------------------

TVQH=THETA(11)*ALLMCL_FFM_HEP ; PLASMA FLOW RATE recommended to be fixed to 90L/h

TVFU=THETA(12) ; UNBOUND PLASMA FRACTION OF isoniazid

; Typical values of CSF penetration parameters--------------------------------------------------------------------------

TVKE0 = THETA(15)

TVPPC = THETA(16)

;----------------------------------------------------------------------------------------------------------------------

; Defining parameters--------------------------------------------------------------------------------------------------

CLINT = TVCL*EXP(BSVCL+BOVCL) ; CLEARANCE (CL now represents intrinsic clearance rather than oral clearance; therefore, initial clearance estimates (THETAs) were set as Oral_CL / fu)

V = TVV*EXP(BSVV) ; CENTRAL VOL.

KA = TVKA*EXP(BSVKA+BOVKA) ; ABS. RATE CONSTANT

BIO = TVBIO*EXP(BSVBIO+BOVBIO) ; BIOAVAILABILITY

V3 = TVV3*EXP(BSVV3) ; PERIPH VOL

Q = TVQ*EXP(BSVQ) ; INTER COMPT CL

MTT = TVMTT*EXP(BOVMTT) ; MTT TIME

NN = TVNN ; Number of transit compartments

QH = TVQH ; PLASMA FLOW RATE recommended to be fixed to 90L/h

FU = TVFU

;Effect parameters

KE0 = TVKE0 * EXP(BSVKE0) ; Plasma-to-CSF equilibration first-order rate constant

PPC = TVPPC * EXP(BSVPPC) ; CSF-to-plasma pseudo-partition coefficient

;----------------------------------------------------------------------------------------------------------------------

; re-parameterization

K32 = Q/V3 ; from peripheral to central

K23 = Q/V ; from central to peripheral

; Transfer constants for hepatic extraction model--------------------------------------------------------------------------------------------------

EH = (CLINT*FU)/((CLINT*FU)+QH) ; fraction undergoing first pass extraction

FH = 1 - EH ; fraction available after 1st pass to go to systemic circulation

K20 = QH*EH /V ; liver elimination rate constant

CL = CLINT

;----------------------------------------------------------------------------------------------------------------------

; Transit compartment absorption

F1=0

KTR = (NN+1)/MTT

IF (NEWIND/=2.OR.EVID>=3) THEN

TNXD=TIME

PNXD=AMT

TIMEDOSE = TIME

AMOUNTDOSE = AMT

ENDIF

TDOS=TNXD

PD=PNXD

IF(AMT>0) THEN

TNXD=TIME

PNXD=AMT

ENDIF

PIZZA = LOG(BIO*PD*KTR + 1E-12) - GAMLN(NN+1)

;----------------------------------------------------------------------------------------------------------------------

A_0(1) = 1E-12; ABS CMT

A_0(2) = 1E-12; CENTRAL CMT

A_0(3) = 1E-12; PERI1 CMT

A_0(4) = 1E-12; CSF CMT

;----------------------------------------------------------------------------------------------------------------------

$DES

C2 = A(2)/V

TEMPO = T-TDOS

KTT = 0

TRANSIT = 0

IF(PD.GT.0.AND.TEMPO.GT.0) THEN

KTT = KTR*(TEMPO)

TRANSIT = EXP(PIZZA+NN*LOG(KTT)-KTT)

ENDIF

DADT(1) = TRANSIT - KA*A(1)

DADT(2) = KA*A(1)*FH - K20*A(2) - K23*A(2) + K32*A(3)

DADT(3) = K23*A(2) - K32*A(3)

DADT(4) = KE0*(PPC*C2 - A(4))

;----------------------------------------------------------------------------------------------------------------------

$ERROR

; Error model for plasma observations----------------------------------------------------------------------------------

LLOQ_P = 0.105

CENS_THR_P = LLOQ_P

CP = A(2)/V

IPRED_P = CP

PROP_P = IPRED_P*THETA(13)

ADD_P = THETA(14)+(CENS_THR_P*0.2)

IF (ICALL/=4.AND.CENS==1.AND.DVID==1) THEN

ADD_P = ADD_P +(LLOQ_P*0.5)

ENDIF

IF (ICALL/=4.AND.CENS==2.AND.DVID==1) THEN

PROP_P = 0

ADD_P = 10000000000

NO_FIT = 1

ENDIF

W_P= SQRT((ADD_P)**2+(PROP_P)**2)

; Error model for CSF observations----------------------------------------------------------------------------------

LLOQ_E = 0.0586

CENS_THR_E = LLOQ_E

CE = A(4)

IPRED_E = CE

PROP_E = IPRED_E*THETA(17)

ADD_E = THETA(18) + (0.2*CENS_THR_E)

IF(ICALL/=4.AND.CENS==1.AND.DVID==4) THEN

ADD_E = ADD_E + (LLOQ_E*0.5)

ENDIF

W_E = SQRT((ADD_E)**2 + (PROP_E)**2)

ERROR_P = W_P * ERR(1)

ERROR_E = W_E * ERR(1)

; Redefine IPRED, residual error weighting, and error term based on DVID--------------------------------------------

IPRED = IPRED_P

W = W_P

ERROR_TERM = ERROR_P

IF(DVID==4) THEN

IPRED = IPRED_E

W = W_E

ERROR_TERM = ERROR_E

ENDIF

; Protective code-----------------------------------------------------------------------------------------------------------------------------------------------------

IF (W.LE.0.000001) W=0.000001

IRES=DV-IPRED

IWRES=IRES/W

Y = IPRED + ERROR_TERM

; To prevent simulation (ICALL==4) of negative values--------------------------------------------------------------

IF (DVID==1.AND.ICALL==4.AND.Y<=LLOQ_P) Y=LLOQ_P/2

IF (DVID==4.AND.ICALL==4.AND.Y<=LLOQ_E) Y=LLOQ_E/2

; To calculate time after dose-------------------------------------------------------------------------------------

IF(AMT>0) THEN

TIMEDOSE = TIME

AMOUNTDOSE = AMT

ENDIF

TAD = TIME-TIMEDOSE

TSOD = TAD

VARCL = BSVCL + BOVCL

VARBIO = BSVBIO + BOVBIO

VARAUC = BSVBIO + BOVBIO - BSVCL - BOVCL

VARABS = BOVKA + BSVKA -BSVMTT - BOVMTT ;BSVLAG - BOVLAG

; Retrieve amount in each compartment-----------------------------------------------------------------------------

AA1 = A(1)

AA2 = A(2)

AA3 = A(3)

AA4 = A(4)

;-------------------------------------------------------------------------------------------------------------------

; Initial estimates ------------------------------------------------------------------------------------------------

$THETA

(15.4) ; 1 CL [L/h] slow

(34.2) ; 2 CL [L/h] intermediate

(68.2) ; 3 CL [L/h] fast

(43.7) ; 4 V [L]

(2.23) ; 5 KA [1/h]

(1) FIX ; 6 BIO

(22.7) ; 7 V3 [L]

(5.1) ; 8 Q [L/h]

(0.255) ; 9 MTT

(5.82) ; 10 NN []

(90) FIX ; 11 QH (L/h)

(0.95)FIX ; 12 FU(%)

(0.164) ; 13 PROP [%]

(0) FIX ; 14 ADD [mg/L]

(0.177) ; 15 KE0 [1/h]

(1.02) ; 16 PPC []

(0.588) ; 17 PROP_E [%]

(0) FIX ; 18 ADD_E [mg/L]

$OMEGA BLOCK(1)

0.0507 ; 1 BSV CL

$OMEGA BLOCK(1)

0 FIX ; 2 BSV V

$OMEGA BLOCK(1)

0 FIX ; 3 BSV KA

$OMEGA BLOCK(1)

0 FIX ; 4 BSV BIO

$OMEGA BLOCK(1)

0 FIX ; 5 BSVV3

$OMEGA BLOCK(1)

0 FIX ; 6 BSVQ

$OMEGA BLOCK(1)

0 FIX ; 7 BSVMTT

$OMEGA BLOCK(1)

0 FIX ; 8 BSVKE0

$OMEGA BLOCK(1)

0 FIX ; 9 BSVPPC

;-------------------------------------------------------------------------------------------------------------------

$OMEGA BLOCK(1)

0 FIX ; 10 BOVCL

$OMEGA BLOCK(1) SAME

$OMEGA BLOCK(1) SAME

$OMEGA BLOCK(1) SAME

$OMEGA BLOCK(1) SAME

;-------------------------------------------------------------------------------------------------------------------

$OMEGA BLOCK(1)

0.103 ; 15 BOVBIO

$OMEGA BLOCK(1) SAME

$OMEGA BLOCK(1) SAME

$OMEGA BLOCK(1) SAME

$OMEGA BLOCK(1) SAME

;-------------------------------------------------------------------------------------------------------------------

$OMEGA BLOCK(1)

1.96 ; 20 BOVKA

$OMEGA BLOCK(1) SAME

$OMEGA BLOCK(1) SAME

$OMEGA BLOCK(1) SAME

$OMEGA BLOCK(1) SAME

;-------------------------------------------------------------------------------------------------------------------

$OMEGA BLOCK(1)

0.758 ; 25 BOVMTT

$OMEGA BLOCK(1) SAME

$OMEGA BLOCK(1) SAME

$OMEGA BLOCK(1) SAME

$OMEGA BLOCK(1) SAME

;-------------------------------------------------------------------------------------------------------------------

$SIGMA 1 FIX

;-------------------------------------------------------------------------------------------------------------------

$ESTIMATION MSFO=run212.msf METHOD=1 INTER MAXEVAL=9999 PRINT=1 NOABORT

NSIG=3 SIGL=9

NONINFETA=1 ETASTYPE=1

;-------------------------------------------------------------------------------------------------------------------

;$COVARIANCE PRINT=E UNCONDITIONAL

;------------------------------------------------------------------------------------------------------------------

$TABLE ....

;------------------------------------------------------------------------------------------------------------------

**REFERENCE**:

52. Johansson ÅM, Karlsson MO. 2013. Comparison of methods for handling missing covariate data. AAPS Journal 15:1232–1241.
